# The Efficacy and Safety of Nivolumab Plus mFOLFOX6 in Gastric Cancer with Severe Peritoneal Metastasis

**DOI:** 10.3390/jcm13030834

**Published:** 2024-01-31

**Authors:** Yurika Nakayama, Takayuki Ando, Naoki Takahashi, Kenichiro Tsukada, Hiroaki Takagi, Yuno Goto, Atsuko Nakaya, Naokatsu Nakada, Hiroki Yoshita, Iori Motoo, Akira Ueda, Yuko Ueda, Miho Sakumura, Shinya Kajiura, Kohei Ogawa, Ayumu Hosokawa, Ichiro Yasuda

**Affiliations:** 1 Third Department of Internal Medicine, University of Toyama, 2630 Sugitani, Toyama 930-0194, Japan; yurika@med.u-toyama.ac.jp (Y.N.); iori4869@med.u-toyama.ac.jp (I.M.); akira-desu@ymail.plala.or.jp (A.U.); i-yuko@umail.plala.or.jp (Y.U.); aomiho@med.u-toyama.ac.jp (M.S.); d12433@med.u-toyama.ac.jp (S.K.); yasudaic@med.u-toyama.ac.jp (I.Y.); 2 Department of Gastroenterology, Kouseiren Takaoka Hospital, 5-10 Eirakumachi, Takaoka-shi 933-8555, Japan; takahashi03998@kouseisen-ta.or.jp (N.T.); tsukada03226@kouseiren-ta.or.jp (K.T.); 3 Department of Medical Oncology, Toyama Prefectural Central Hospital, 2-2-78 Nishinagae, Toyama-shi 930-8550, Japan; dxgnj151@yahoo.co.jp (H.T.); kogawa@fuga.ocn.ne.jp (K.O.); 4 Department of Gastroenterology, Takaoka City Hospital, 4-1 Takaramachi, Takaoka-shi 933-8550, Japan; yuno.5330@gmail.com (Y.G.); atsuko_nakaya@med-takaoka.jp (A.N.); 5 Department of Gastroenterology, Itoigawa General Hospital, 457-1 Takegahana, Itoigawa-shi 941-8502, Japan; dr-nao@f2.dion.ne.jp; 6 Department of Gastroenterology, Toyama Nishi General Hospital, 1019 Fuchumachi Shimokutsuwada, Toyama-shi 939-2716, Japan; hiroki570205@yahoo.co.jp; 7 Department of Clinical Oncology, University of Miyazaki Hospital, Kihara-5200 Kiyotakecho, Miyazaki-shi 889-1692, Japan; ayhosoka@med.miyazaki-u.ac.jp

**Keywords:** gastric cancer, severe peritoneal metastasis, mFOLFOX6, nivolumab

## Abstract

(1) **Background**: Nivolumab plus chemotherapy is established as a first-line treatment for advanced gastric cancer (AGC). While mFOLFOX6 is commonly used for AGC with severe peritoneal metastasis, the efficacy of nivolumab combined with it remains uncertain. We evaluated the outcomes of nivolumab plus mFOLFOX6 for AGC with severe peritoneal metastasis in clinical practice. (2) **Methods**: This multicenter retrospective study was conducted between December 2021 and June 2023. We investigated AGC patients with massive ascites or inadequate oral intake due to severe peritoneal metastasis and who received nivolumab plus mFOLFOX6. (3) **Results**: Among 106 patients treated with nivolumab plus chemotherapy, 21 (19.8%) had severe peritoneal metastasis, with 14 receiving nivolumab plus mFOLFOX6. The median progression-free survival was 7.4 months (95%CI 1.9-10.1), and the median overall survival was 10.7 months (95%CI 5.3-NA), with four patients (28.5%) surviving more than 12 months. Improved ascites and oral intake were observed in 6/14 patients (42.8%) and 10/11 patients (90.9%), respectively. The major grade 3 or more adverse events included leukopenia (28.5%) and neutropenia (21.4%), with no severe immune-related adverse events reported. (4) **Conclusions**: The safety and moderate efficacy of nivolumab plus mFOLFOX6 were suggested even in AGC patients with severe peritoneal metastasis.

## 1. Introduction

Gastric cancer is common in East Asia, accounting for more than 60% of cases [1]. In Japan, approximately 40,000 people die annually of gastric cancer, making it the second leading cause of cancer death [2]. Although the age-adjusted morbidity and mortality rates of gastric cancer have decreased in recent years because of the decrease in *Helicobacter pylori* infection and advances in testing and treatment [3,4], the number of gastric cancer patients and deaths is still increasing because of the aging society [5], and the 5-year survival rate remains poor at 20–40% [6]. In gastrointestinal malignancies, peritoneal metastasis frequently occurs during progression [7,8] and was detected in 14% of gastric cancer cases at the time of initial diagnosis in a registry study from the Netherlands [9]. Although peritoneal metastasis is a poor prognostic factor, patients with the condition have often been excluded from pivotal clinical trials because of tumor-related complications, and treatment outcomes for severe peritoneal metastasis have not been clarified.

In Japan, combination therapy with S-1 or capecitabine and cisplatin or oxaliplatin is the first-line standard regimen for advanced gastric cancer (AGC) [10,11,12]. However, oral fluoropyrimidine and cisplatin are unacceptable in patients with AGC with renal dysfunction or bowel obstruction due to peritoneal metastasis. Although continuous infusion of 5-fluorouracil (5-FU ci) is the standard chemotherapy regimen for AGC with peritoneal metastasis according to the JCOG0106 trial [13], hospitalization is required each time. On the other hand, the ISO-5FU study demonstrated that a weekly bolus of 5-FU/l-leucovorin (l-LV) was noninferior to S-1, and the JCOG9912 trial showed that S-1 was noninferior to 5-FU ci [10]. Therefore, a bolus of 5-FU/l-LV is most often administered to patients with AGC who have severe peritoneal metastasis and massive ascites or inadequate oral intake. Furthermore, 5-FU/l-LV plus paclitaxel (FLTAX) treatment for such patients showed an ascites response rate of 44%, the median progression-free survival (PFS) of 4.2 months, and median overall survival (OS) of 8.0 months [14]. Based on these results, a phase II/III trial (JCOG1108/WJCOG7312G) was conducted in Japan, which compared FLTAX with 5-FU/l-LV for AGC patients with severe peritoneal metastasis. Although FLTAX did not show significant superiority to 5-FU/l-LV in terms of OS [median OS, 7.3 vs. 6.1 months; hazard ratio (HR) 0.79, confidence interval (CI) 0.60–1.05; *p* = 0.14]. On the other hand, PFS [median OS, 5.4 vs. 1.9 months; HR 0.64, CI 0.43–0.96; *p* = 0.029] and QOL outcomes were favorable [15].

5-FU/l-LV plus oxaliplatin (FOLFOX) is one of standard regimens for AGC patients. This regimen can be administered to patients with severe peritoneal metastasis because it contains only intravenous infusion and does not require hydration. In addition, FOLFOX has been shown to be effective and feasible for AGC with a poor PS. The incidence of grade 3 or higher neutropenia associated with FOLFOX was 17% in patients with PS 2 [16], which was comparable to that observed in PS 0 or 1 patients, (29–45%) [17,18,19], and the response rate of FOLFOX was 32% in patients with PS 2 [16]. According to a phase II trial of mFOLFOX4 for AGC with ascites, the ascites response was observed in 35.4% of patients [20]. Thus, FOLFOX seems promising for patients with severe peritoneal metastasis or those with a poor PS. Based on these findings, two retrospective studies of mFOLFOX6 for AGC patients with severe peritoneal metastasis were conducted, and they demonstrated that OS was 8.8 to 13.2 months associated with a decrease in ascites and improvement in oral intake [21,22].

Recently, nivolumab plus chemotherapy, including nivolumab plus FOLFOX, has been established as one of the first-line treatments for HER2 negative gastric cancer based on the ATTRACTION-4 [23] and CheckMate 649 trials [24]. Indeed, it has been suggested that the anti-tumor efficacy of oxaliplatin is attributed to the induction of immunogenic cell death (ICD), which contributes to anti-tumor immunity by releasing damage-related molecular patterns (DAMPs) [25,26,27]. Several studies have demonstrated a synergetic interaction between oxaliplatin and PD-1/PD-L1 inhibitors [28,29,30], and additional benefits of nivolumab when combined with FOLFOX were expected. Therefore, adding immune checkpoint inhibitors to FOLFOX from first-line treatment is expected to improve therapeutic effects even in patients with severe peritoneal metastasis. However, the outcome of nivolumab in combination with FOLFOX remains unclear in AGC patients with these conditions.

This study aimed to evaluate the efficacy and safety of nivolumab plus mFOLFOX6 as a first-line treatment for AGC patients with severe peritoneal metastasis.

## 2. Materials and Methods

### 2.1. Study Design and Patients

We conducted a multicenter retrospective analysis at seven institutions, including University of Toyama (Toyama, Toyama, Japan), Toyama Prefectural Central Hospital (Toyama, Toyama, Japan), Takaoka City Hospital (Takaoka, Toyama, Japan), Kouseiren Takaoka Hospital (Takaoka, Toyama, Japan), Toyama Nishi General Hospital (Toyama, Toyama, Japan), Itoigawa General Hospital (Itoigawa, Niigata, Japan), and University of Miyazaki Hospital (Miyazaki, Miyazaki, Japan) between December 2021 and June 2023. Patients with severe peritoneal metastasis who received nivolumab plus mFOLFOX6 regimen were enrolled in this study according to the following eligibility criteria: (1) histologically confirmed gastric or gastroesophageal adenocarcinoma, (2) unresectable or recurrent disease, (3) massive ascites and/or inadequate oral intake due to severe peritoneal metastasis, (4) treatment with nivolumab plus mFOLFOX6 regimen, (5) no previous chemotherapy, except adjuvant chemotherapy finished more than 6 months before the starting date of nivolumab plus mFOLFOX6, and (6) no previous treatment with oxaliplatin.

Severe peritoneal metastasis was defined as the presence of massive ascites and/or inadequate oral intake due to peritoneal metastasis. Ascites were assessed through computed tomography (CT) scans before treatment, and classified as follows: massive ascites extended from the pelvic cavity to the upper abdomen, mild ascites were limited to the pelvic cavity or the upper abdomen, and moderate ascites were between massive and mild. Inadequate oral intake due to severe peritoneal metastasis was identified in patients requiring continuous intravenous infusion. The diagnosis of peritoneal metastasis was confirmed when a CT scan revealed obvious peritoneal nodules, ascites, hydronephrosis, increased concentration of peritoneal fatty tissue, thickening of the bowel wall, and obstruction of the bile duct, excluding factors other than peritoneal metastasis. 

We reviewed medical records, which included sex, age, European Cooperative Oncology Group (ECOG) PS, disease status (advanced or recurrent), history of gastrectomy, histology type, human epidermal growth factor receptor 2 (HER2) status, microsatellite instability (MSI) status, PD-L1 combined positive score (CPS) status, ascites status, oral intake status, metastatic sites, and number of metastatic sites.

This study was approved by the institutional review boards of each participating institute, including the Toyama University Hospital (ethic code: R2023195). This research was conducted ethically in accordance with the World Medical Association Declaration of Helsinki. An opt-out approach, which was approved by Review Committee, was used for informed consent.

### 2.2. Treatments

The nivolumab plus mFOLFOX6 regimen consisted of nivolumab (240 mg) administered over 30 min and oxaliplatin (85 mg/m^2^) with leucovorin (200 mg/m^2^) given simultaneously over 2 h, followed by a bolus of 5-FU (400 mg/m^2^) and a continuous infusion of 5-FU (2400 mg/m^2^) for 46 h. Treatment was continued until disease progression, the occurrence of unacceptable toxicity, cancer remission, or a patient’s decision to discontinue the therapy. The 5-FU or oxaliplatin dose was reduced because of old age or poor PS, or in the case of grade 4 hematological or grade 3–4 nonhematological adverse events.

Relative dose intensity (RDI) was calculated for each patient as the ratio of delivered to planned chemotherapy dose intensity. The ratio was determined by dividing the total dosage that the patient received for each drug within each regimen by the total dosage specified by the corresponding standard regimen.

### 2.3. Assessments and Statistical Analysis

Tumor response was assessed using CT imaging and the Response Evaluation Criteria in Solid Tumors (RECIST) version 1.1. The objective response rate (ORR) was defined as the proportion of patients with complete response (CR) or partial response (PR) among those with target lesions. The disease control rate (DCR) was defined as the proportion of patients with CR, PR, or stable disease (SD). Patients without measurable lesions were excluded from the response rate analysis. PFS was defined as the period from the first administration of chemotherapy to the radiological or clinical observation of disease progression or death from any cause. OS was defined as the time from the first administration of chemotherapy to death from any cause. The response in ascites was also evaluated. The best response to ascites was defined as “ascites CR” when ascites completely disappeared, “ascites PR” when ascites levels decreased, “ascites SD” when ascites were at the same level as before treatment, and “ascites progressive disease (PD)” when ascites levels increased [15]. Improvement in oral intake was defined as withdrawal from continuous intravenous infusion for more than a week after initiating nivolumab plus mFOLFOX6. The change in tumor burden was assessed using CT scans every two months, with additional scans performed at the discretion of the attending doctor. Toxicity and immune-related adverse events (irAEs) were graded according to the Common Terminology Criteria for Adverse Events (CTC-AE) ver. 5.0. Treatment-related death was also evaluated. PFS and OS were estimated using the Kaplan–Meier method and compared using the log-rank test. All statistical analyses were performed using EZR version 1.54 (https://www.jichi.ac.jp/saitama-sct/SaitamaHP.files/statmedOSX.html, accessed on 22 January 2023), and *p* ≤ 0.05 was considered to indicate statistically significant differences.

## 3. Results

### 3.1. Patient Characteristics and Treatment Exposure

**Figure 1:**
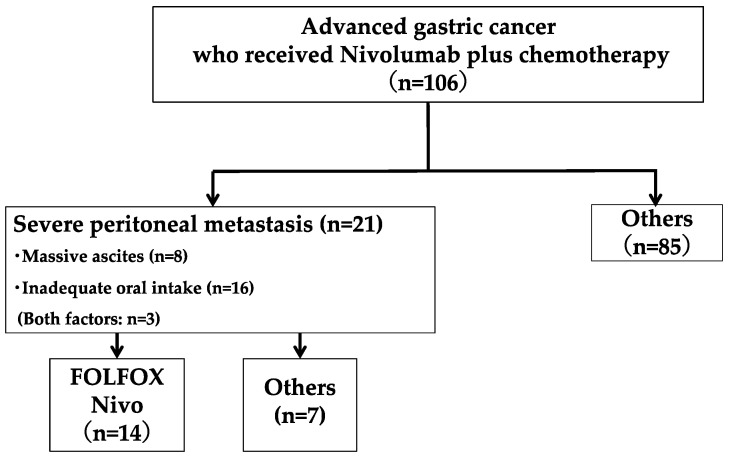
CONSORT flow diagram.

A total of 106 patients with AGC were treated with nivolumab plus chemotherapy. Among them, 21 (19.8%) had massive ascites or inadequate oral intake because of severe peritoneal metastasis, and 14 received nivolumab plus mFOLFOX6 and 7 received other chemotherapy regimens, including S-1 and oxaliplatin (SOX) or capecitabine and oxaliplatin (XELOX) (Figure 1). All of them fulfilled the eligibility criteria for this study. The patients’ characteristics are shown in Table 1. The median age was 72 years (range, 56–82). No patient had an ECOG PS of 2. Three patients had a history of gastrectomy: one had a total gastrectomy and two had distal gastrectomies (one due to pyloric stenosis). Nine patients had tumors expressing a PD-L1 combined positive score (CPS) < 5, and one had a CPS ≥5. Among all the patients, six had massive ascites, and eleven had inadequate oral intake. Three patients had massive ascites and inadequate oral intake.

Dose intensity is presented in Table 2. The median number of treatment cycles for mFOLFOX and nivolumab were 8 (range 2–19 cycles) and 6 (range 1–18 cycles), respectively. Seven patients received nivolumab from the first course. Patients receiving nivolumab after the second cycle were determined by the attending physician, based on the confirmation of a HER2 status and concerns about toxicity. Dose modification from the first cycle was performed in seven patients: three due to age, two due to PS, one due to post-myelotoxicity, and one due to hemodialysis. The median RDI was 75.0% (range 45–100%) for bolus 5-FU, 83.3% (range 60–100%) for 5-FU continuous infusion, 76.1% (range 25.5–100%) for oxaliplatin, and 94.9% (range 7.1–100%) for nivolumab. Three patients discontinued oxaliplatin: two due to peripheral neuropathy (Cases 1 and 9) and one due to an allergic reaction (Case 2). Nivolumab was discontinued in one patient because of irAEs (Grade 2 erythema multiforme, Case 2).

A total of 106 advanced gastric cancer patients were treated with nivolumab plus chemotherapy. Among them, 21 (19.8%) had severe peritoneal metastasis (8 patients had massive ascites, 16 had inadequate oral intake, and 3 of them had both factors). Fourteen of them received nivolumab plus mFOLFOX6, and seven patients received other chemotherapy (nivolumab plus SOX or XELOX).

### 3.2. Efficacy

**Figure 2:**
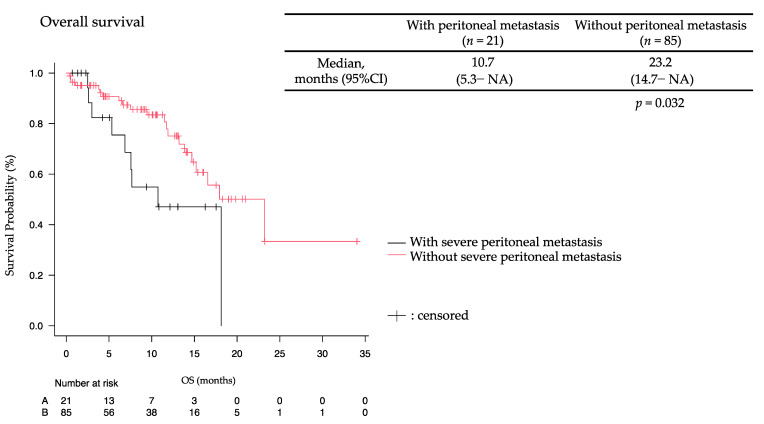
Overall survival in advanced gastric cancer patients with severe peritoneal metastasis (*n* = 21) and without severe peritoneal metastasis (*n* = 85). NA: not analyzed.

**Figure 3:**
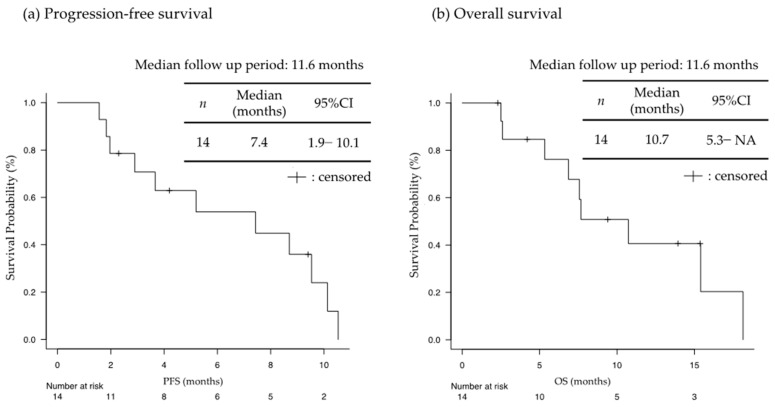
(**a**) Progression-free survival and (**b**) overall survival in patients with severe peritoneal metastasis who received nivolumab plus mFOLFOX6 regimen (*n* = 14). NA: not analyzed.

In 106 patients with AGC, patients with severe peritoneal metastasis (*n* = 21) exhibited a median OS of 10.7 months (95%CI: 5.3–NA), while those without peritoneal metastasis (*n* = 85) had a median OS of 23.2 months (95%CI: 14.7–NA) (*p* = 0.032), with a median follow-up period of 8.9 months (Figure 2). Among patients with severe peritoneal metastasis, 14 patients who received nivolumab plus mFOLFOX6 had a median PFS (Figure 3a) and OS (Figure 3b) of 7.4 months (95%CI 1.0–10.1) and 10.7 months (95%CI 5.3–NA), respectively, with a median follow-up period of 11.6 months. Additionally, there were two patients with favorable OS (15.4 and 18.1 months). On the other hand, seven patients who received other chemotherapy (SOX or XELOX) had a median PFS of 7.9 months (95%CI 1.8–NA), and a median OS was not yet reached. 

Among 10 patients with a target lesion who were treated with nivolumab plus mFOLFOX6 regimen, 3 patients achieved PR, resulting in an ORR of 30.0%. Five patients had SD, leading to a DCR of 80.0% (Table 3). Regarding ascites response, one patient achieved ascites CR and three achieved ascites PR, respectively, resulting in a response rate of 42.8% (Table 3). Additionally, improvement in oral intake was observed in 10/11 (90.9%) with inadequate oral intake (Table 4). 

Nivolumab plus mFOLFOX6 was discontinued in 11 patients because of disease progression. Among them, seven patients (63.6%) received second-line chemotherapy, and four patients did not receive any chemotherapy and were treated with the best supportive care. In the second-line treatment, five patients were treated with nab-paclitaxel/paclitaxel and ramucirumab combination chemotherapy, and two patients received nab-paclitaxel/paclitaxel monotherapy (Table 4).

### 3.3. Adverse Events

The major grade 3 or 4 adverse events were leukopenia (28.5%), neutropenia (21.4%), anemia (7.1%), decreased appetite (7.1%), and diarrhea (7.1%). However, the frequencies of grade 3 or higher were relatively low (Table 5). Although death within 30 days after discontinuation of chemotherapy was seen in three patients, the patients died of worsening disease after being transferred to the best supportive care. IrAEs were observed in two patients with arthritis (Case 1) and erythema multiforme (Case 2), respectively (Table 6); arthritis was untreated, and erythema multiforme was treated with prednisolone ointment. Oral prednisolone was not required in any patient. Although the symptoms improved in the patient with erythema multiforme after discontinuing nivolumab and ointment, nivolumab was not resumed. During the withdrawal of nivolumab, chemotherapy was continued. Severe irAEs did not occur, regardless of the PD-L1 status.

## 4. Discussion

We evaluated the efficacy and safety of nivolumab plus chemotherapy as a first-line treatment for AGC patients with severe peritoneal metastasis in clinical practice. Although several studies have reported outcomes with FOLFOX, to the best of our knowledge, this is the first report of nivolumab combined with chemotherapy in patients with severe peritoneal metastasis. 

According to the Clinical Practice Guidelines for Peritoneal Malignancy 2021 [31], patients with severe peritoneal metastasis often have a poor general condition and should be carefully selected for chemotherapy, considering the best supportive care. However, two retrospective studies of mFOLFOX6 reported prolonged PFS of 4.2–7.5 months and OS of 8.8–13.2 months [21,22]. In this study, median PFS and OS were 7.4 months and 10.7 months, respectively, which are comparable to the reported results of FOLFOX (Table 7). Importantly, the oral intake improved in a large population of patients, and four patients (28.5%) demonstrated survival beyond 12 months (Table 7). These promising findings suggest that specific patients may experience prolonged efficacy, although the additional benefit of nivolumab was not evident in the entire population. Notably, the OS in patients without severe peritoneal metastasis was 23.2 months, indicating that the management skill and judgment of the attending physicians for AGC seemed appropriate. 

One possible reason the median PFS and OS in our study were not as long as expected is the small number of patients with CPS ≥ 5, although the proportion of AGC patients with CPS ≥ 5 has been reported to be 60% [24]. Indeed, the addition of the immune checkpoint inhibitor (ICI) to chemotherapy showed limited survival benefits in AGC with low or negative PD-L1 expression [32,33]. In particular, some reports showed that the efficacy of ICI was limited in patients with ascites. Kaneko et al. [34] demonstrated that nivolumab is distributed into ascites, and the retention of ascites, and its removal may result in decreased systemic drug exposure to nivolumab. The decreased survival benefit of nivolumab combined with chemotherapy in this study could be attributed to the reduced efficacy of nivolumab in patients with massive ascites. In addition, Fuca et al. [35] reported that patients with metastatic MSI-high or mismatch repair deficient (MMR-D) gastrointestinal cancers and peritoneal metastasis, who had ascites, exhibited worse outcomes with ICI therapy compared to patients with peritoneal metastasis without ascites or patients without peritoneal metastasis. Although these results were obtained with MSI high or MMR-D, the same would be expected for microsatellite stable (MSS) gastric cancer with peritoneal metastasis, and, in our study, the effect of ICI was possibly limited. Furthermore, resistance to systemic ICI in patients with malignant ascites has been reported [36,37,38,39]. The peritoneum is isolated from the systemic circulation by the peritoneum plasma barrier, limiting access to chemotherapy and the immune system. Although ICI generally shows efficacy in MSI-high tumors, peritoneal metastasis may demonstrate resistance that arises from factors related to tumor characteristics, the immunosuppressive state of the peritoneal cavity, paracrine factors within malignant ascites, or tumor–peritoneum interactions. For example, the ascites of patients with AGC exhibited a high proportion of T lymphocytes with CD69 or PD-1, memory T cells marked with CD45RO, and an increased number of Foxp3+ T regulatory cells [40]. Furthermore, ascites of patients with peritoneal metastasis contained significantly higher levels of IL-10, TGFb1, TGFb2, and TGFb3 than serum [41]. Indeed, the proportion of CD8 T lymphocytes with memory and activation markers (HLA-DR), CD3 T lymphocytes with PD-1, and the number of FoxP3+ Tregs were identified as independent prognostic factors [40]. In addition, Chow A et al. showed that macrophages expressing high levels of Tim-4+ in the serous body cavities contributes to reduced numbers of CD8+ T cells with tumor-reactive features. Therefore, immunotherapy can be limited in patients with peritoneal metastasis exhibiting these microenvironments [42]. These studies suggest that diverse immune cells are widely distributed, and immune profiling of ascites is necessary for selecting benefits even if ICI is used in addition to chemotherapy. 

In this study, half of the patients received nivolumab after the second course of chemotherapy because of the possibility of adverse events. The transition rate to second-line chemotherapy was 63.6%, similar to the 69–85% in Japanese clinical trials [43]. The treatment dose was modified for all patients in our study; however, there were a few delays due to adverse events, and RDI was relatively maintained. The results showed that nivolumab with chemotherapy can be safely administered to patients with massive ascites or inadequate oral intake due to severe peritoneal metastasis. Some studies have reported that combination therapy of vascular endothelial growth factor (VEGF)/vascular endothelial growth factor receptor (VEGFR) inhibitor with ICI had a synergistic and improved antitumor effect [44,45]. In this study, four patients who received second-line treatment showed a response for a relatively long time after discontinuation of nivolumab, suggesting that the effect of ICI may have continued after nivolumab was discontinued. Although the number of patients who transitioned to second-line treatment is small and the follow-up period was short, the outcome of second-line treatment after ICI should be investigated.

Several studies have demonstrated an association between irAEs and the efficacy of ICI [46,47,48,49]. In 32 AGC patients with moderate to massive ascites treated with ICI monotherapy, the response to effusion was 50.0% (3/6) in patients with irAEs and 7.6% (2/26) in patients without irAEs [50]. In our study, the two cases that developed irAEs had the longest OS (15.4 and 18.1 months, respectively), and similar efficacy can be inferred for patients with severe peritoneal metastasis.

Several limitations of the study need to be acknowledged. First, the study was retrospective, and the follow-up period for OS might have been insufficient. Additionally, the sample size was small. The reason for the small sample size was that most patients with massive ascites or inadequate oral intake due to severe peritoneal metastasis were unlikely to receive chemotherapy because of their poor PS and the possibly high incidence of adverse events. CPS was also not measured in some patients, and the study of outcomes with PD-L1 expression is insufficient.

## 5. Conclusions

Our study indicates that nivolumab plus mFOLFOX6 is safe and moderately effective in AGC patients with severe peritoneal metastasis, with certain patients exhibiting long-term survival. However, further investigation is required to assess its efficacy in a larger patient population. 

## Figures and Tables

**Table 1:** Patient characteristics.

		*n* = 14
Sex	Male/Female	8/6
Age (years)	Median (range)	72 (56–82)
Performance status (ECOG)	0/1/2	2/12/0
Disease status	Unresectable/Recurrence	12/2
History of gastrectomy	−/+	11/3
Histologic type	Intestinal/Diffuse	6/8
HER2 ^1^ status	−/+	14/0
MSI ^2^ status	MSS/MSI-high/NE	4/0/10
PD-L1 CPS ^3^ status	<5/≥5/NE	9/1/4
Ascites	Mild/Moderate/Massive	6/2/6
Oral intake	Adequate/Inadequate	3/11
Metastatic sites	Lymph node/Liver/Peritoneal	11/3/14
Number of metastatic sites	1−2/≥3	10/4

^1^ HER2: Human epidermal growth factor receptor 2. ^2^ MSI: microsatellite instability. ^3^ CPS: PD-L1 combined positive score.

**Table 2:** Treatment exposure.

Case	Age	PS	Ascites	Oral Intake	Initial Dose (mg/m^2^)			Total Course		Relative Dose Intensity (%)			
					5-FU (b)	5-FU (ci)	L-OHP	mFOLFOX	Nivo	5-FU (b)	5-FU (ci)	L-OHP	Nivo
1	56	1	Mild	Inadequate	400	2400	85	18	13	76.4	84.3	31	72
2	68	1	Mild	Inadequate	400	2400	85	15	7	53.3	67.7	25.5	45
3	67	1	Mild	Inadequate	400	2400	85	4	4	81.3	87.5	82.3	100
4	68	1	Massive	Adequate	200	1600	85	10	10	45	60	87.6	95
5	68	1	Massive	Inadequate	400	2400	85	9	6	90.2	90.2	71.6	57
6	74	1	Moderate	Inadequate	400	2400	65	19	18	88.2	91.2	30.9	94.7
7	48	1	Massive	Adequate	400	2400	85	4	4	87.5	88.9	87.7	91.7
8	72	1	Massive	Inadequate	200	1600	50	14	1	47.6	63.5	56	7.1
9	65	1	Massive	Adequate	400	2400	85	11	10	100	100	75.9	91
10	77	1	Massive	Inadequate	400	2400	85	7	7	75	81.7	76.2	95.2
11	72	1	Moderate	Inadequate	300	2000	85	5	5	75	83.3	100	100
12	74	1	Mild	Inadequate	300	2000	65	2	2	75	83.3	76.5	100
13	77	1	Mild	Inadequate	200	1600	50	6	6	50	66.6	58.8	100
14	82	1	Mild	Inadequate	300	2000	65	2	2	75	83.3	76.5	100

b: bolus, ci: continuous infusion, L-OHP: Oxaliplatin, Nivo: Nivolumab.

**Table 3:** Response for the target lesion and ascites in patients who received nivolumab plus mFOLFOX6 regimen (*n* = 14).

	CR	PR	SD	PD	Response Rate (%)	Disease Control Rate (%)
Response for the target lesion (*n* = 10)	0	3	5	2	30% (3/10)	80% (8/10)
Response for ascites (*n* = 14)	1	5	8	0	42.8% (6/14)	

**Table 4:** Efficacies, PFS, and OS in the study patients.

Case	Ascites	Ascites Response	Oral Intake	Improved Oral Intake	Tumor Response	PFS (Months)	2nd Line	2nd Line PFS (Months)	OS (Months)
1	Mild	PR	Inadequate	Yes	SD	10.5	PTX	7.6	18.1
2	Mild	SD	Inadequate	Yes	SD	8.7	PTX+Ram	6.7	15.4
3	Mild	SD	Inadequate	Yes	PD	1.8	PTX+Ram	13.5 *	15.3 *
4	Massive	SD	Adequate	-	nonCR/nonPD	9.5	nabPTX+Ram	4.4 *	13.9 *
5	Massive	PR	Inadequate	Yes	SD	10.1	-	-	10.7
6	Moderate	CR	Inadequate	Yes	PR	9.4 *	-	-	9.4 *
7	Massive	SD	Adequate	-	nonCR/nonPD	2.9	nabPTX+Ram	4.7	7.6
8	Massive	PR	Inadequate	Yes	PR	7.4	-	-	7.5
9	Massive	PR	Adequate	-	SD	5.2	nabPTX	1.6	6.8
10	Massive	SD	Inadequate	Yes	SD	3.6	PTX+Ram	1.6	5.3
11	Moderate	PR	Inadequate	Yes	PR	1.9	-	-	2.6
12	Mild	SD	Inadequate	No	PD	1.5	-	-	2.5
13	Mild	SD	Inadequate	Yes	SD	2.3 *	-	-	2.3 *
14	Mild	SD	Inadequate	Yes	nonCR/nonPD	1.7 *	-	-	1.7 *

CR: complete response, PR: partial response, SD: stable disease, PD: progressive disease, PFS: Progression-free survival, OS: overall survival, *: censored date, PTX: paclitaxel, nabPTX: nab-paclitaxel, Ram: ramucirumab.

**Table 5:** Adverse events (*n* = 14).

Grade	Any (%)	1–2	3–4
Leukopenia	8 (57.1)	4	4
Neutropenia	8 (57.1)	5	3
Fever neutropenia	0 (0)	0	0
Anemia	9 (64.3)	8	1
Thrombocytopenia	5 (35.7)	5	0
Nausea	7 (50.0)	7	0
Vomiting	4 (28.6)	4	0
Decreased appetite	9 (64.3)	8	1
Fatigue	8 (57.1)	8	0
Diarrhea	2 (14.3)	1	1
Constipation	4 (28.6)	4	0
Peripheral neuropathy	9 (64.3)	9	0

**Table 6:** Immune-related adverse events (*n* = 14).

Grade	Any (%)	1–2	3
All	2 (14.3)	2	0
Arthritis	1 (7.1)	1	0
Erythema multiforme	1 (7.1)	1	0

**Table 7:** Comparison of previous studies and the present study in patients with severe peritoneal metastasis.

	Masuishi et al. [21]	Osumi et al. [22]	This Study
Regimen	mFOLFOX6	mFOLFOX6	Nivolumab+mFOLFOX6
Total number of patients	10	17	14
Median age (range)	64.5 (40–94)	67 (29–74)	72 (56–82)
PFS (median, months)	7.5	4.2	7.4
PFS rate at 6 months (%)	60.0	ND	42.8
OS (median, months)	13.2	8.8	10.7
OS rate at 12 months (%)	50.0	ND	28.5
Improved oral intake (%)	57.0	83.0	90.9
Ascites response (%)	78.0	50.0	42.8
Adverse event (≥Grade3) (%)			
Leukopenia	0	0	28.6
Neutropenia	35.3	30	21.4
Febrile neutropenia	5.9	0	0
Anemia	0	30	7.1
Increased AST ^1^	0	20	0
Increased ALT ^2^	0	20	0
Vomiting	5.9	0	0
Decreased appetite	5.9	0	7.1
Diarrhea	0	0	7.1

^1^ AST: aspartate aminotransferase, ^2^ ALT: alanine aminotransferase, ND: not determined.

## Data Availability

Data are contained within the article.
